# Fingerprint-Based Patient Identification Technology: Simulated Pilot Study on Technical Performance and Usability

**DOI:** 10.2196/88657

**Published:** 2026-07-09

**Authors:** Ginger Schroers, Marta del Rio Oliva, Jenny O'Rourke, Antxon Caballero, Luis Barata Gomes, Raquel Araújo

**Affiliations:** 1 Loyola University Chicago, 2160 S. First Avenue, Maywood, IL, 60153, United States, 1 708-216-9000; 2 Heuristik Technologies S.L., Granada, Spain; 3 Hospital da Luz, Lisboa, Portugal; 4 Risk Management and Quality Division, Luz Saúde, Lisboa, Portugal

**Keywords:** patient identification system, patient tracking, patient safety, healthcare system, medical errors, simulation

## Abstract

This pilot study found that an artificial intelligence–supported fingerprint-based patient identification system demonstrated preliminary technical feasibility and user acceptability among healthcare workers in a simulated clinical environment, warranting further real-world evaluation.

## Introduction

An estimated 1 in 20 patients is exposed to preventable harm, with 12% of harm considered severe or fatal [1]. Patient identification errors are the root cause of many patient safety incidents in both inpatient and outpatient settings [2,3].

Common patient identification methods include person-specific information printed on wristbands or a person’s medical card. Additionally, algorithmic approaches, matching software, radio frequency identification devices, and biometrics may be used. All patient identification methods have limitations to operability [4]. A primary goal of the Joint Commission [5] is to improve the accuracy of patient identification. Innovative approaches are needed to accomplish this goal.

**Figure 1.**
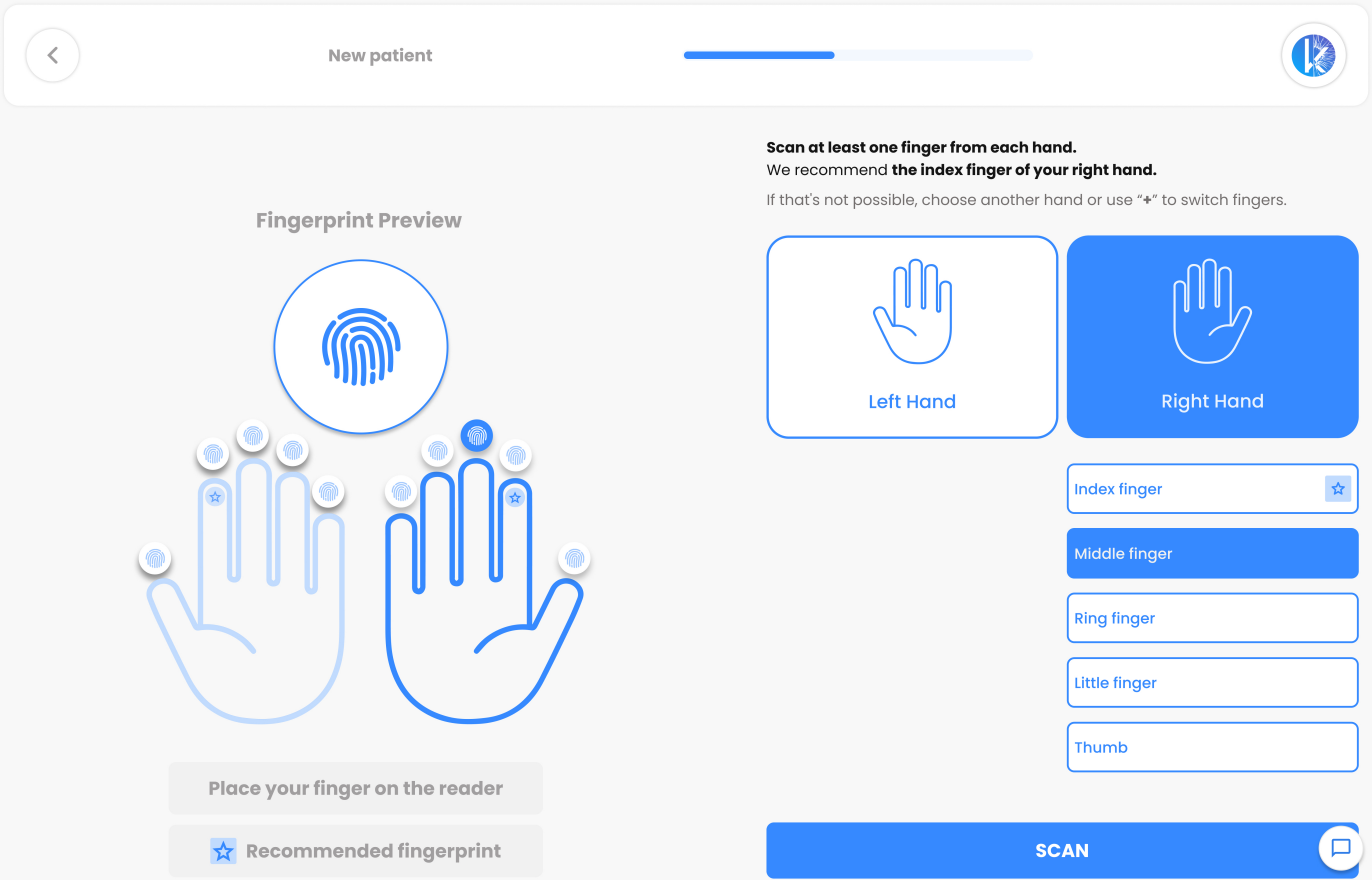
Heuristik interface. The image depicts the user-facing software dashboard displaying the identification verification process.

Fingerprints are individual, nontransferable, and inherent to each person, thus ideal for patient identification. Heuristik utilizes advanced fingerprint identification technology (Figure 1) supported by artificial intelligence to enable identification in adverse situations (wet or dirty fingerprints). Upon admission, consenting patients can opt for fingerprint collection using Heuristik’s biometric software instead of standard identification procedures. Using secure connectivity, the encrypted fingerprints are then sent to a secure cloud-based server for remote processing, where they are pseudonymized and stored. To ensure privacy, Heuristik creates a unique identification method linked to the fingerprint. This method acts as a secure bridge to the patient’s medical record without accessing the patient’s medical record information.

This study investigates the technical feasibility (accuracy) and acceptability (user satisfaction) of the Heuristik system.

## Methods

### Study Design

A simulated multimethods cross-sectional study was conducted in March 2025 at Hospital da Luz’s simulation center in Lisbon, Portugal. The primary objective was to evaluate technical feasibility by matching fingerprints against a pseudonymized database across simulated scenarios. For privacy, Heuristik operated standalone without live electronic health records integration. Participants recruited via purposeful sampling included nurses, technicians, and other health care workers. Eligibility required being >18 years old, providing informed consent, and having usable fingerprints.

The workflow involved a brief training session, then a one-time enrollment phase prior to admission, followed by identification scans at preoperative, postoperative, and outpatient settings. Four Heuristik devices were deployed across scenarios. Before admissions, 12 simulated patients were enrolled using two fingerprints per hand. Researchers used structured forms to manually calculate accuracy by comparing point-of-care scans against registration data.

Usability data were collected via a revised version of the 10-item System Usability Scale tool (SUS) [6], where scores can range from 0‐100 (68 considered as average [7]). Six items were added to the tool to assess usefulness, reliability, relevance, and error reduction on a 5-point scale, plus two open-ended items regarding likes and dislikes. Scale items underwent descriptive analysis in Excel; two researchers content-analyzed open-ended responses.

### Ethical Considerations

The study was approved by the Luz Saúde Investigation Committee (February 20, 2025) and the Hospital da Luz Lisboa Ethics Committee (March 14, 2025; Ref. CES/11/2025/IE). The research adhered to the Helsinki Declaration, and all participants provided written informed consent prior to enrollment.

## Results

Eighteen health care workers with a mean age of 36 (range 24‐50 y, 56% female, 100% Portuguese) participated in the study. The cohort was divided into two groups: 9 health care professionals acting as system testers (5 nurses, 2 patient-care technicians, and 2 client-service technicians) and 9 nonclinical participants ('others’) who acted as simulated patients. Within the professional group, 3 nurses assumed dual roles as both testers and patients, resulting in a total of 12 simulated patients and 6 professional participants who acted solely as system users.

Immediately prior to the clinical workflows, the 12 simulated patients were enrolled using two fingerprints per hand. Subsequent verification confirmed 100% identification accuracy for all participants using a scan of at least one enrolled fingerprint. Upon admission to the simulated settings, client service technicians independently scanned two fingerprints from two different simulated patients. Each patient underwent three scans using alternating hands, totaling six successful identifications with a 100% match rate. A team of six staff members (four nurses and two technicians) performed all subsequent identifications on four patients using bilateral scans. All the scans performed across three clinical settings (outpatient, preoperative, and postoperative) achieved 100% accuracy.

Regarding the acceptability data, seven staff members completed the survey. The SUS scores ranged from 70 to 97.5 (mean 87.14, SD 8.28). All (n=7; 100%) rated the 5-point scale items as “strongly agree” or “agree”. Open-ended responses revealed that participants liked the “reliability” (n=3), “ease of use or practical” (n=3), and “functionality and speed of client identification” (n=1) most about the product. Responses to “What did you like least about the product” included “nothing” (n=2) or were unable to be analyzed (n=5) as the responses did not refer to the product. These results suggest that Heuristik demonstrates high technical accuracy and user acceptability in a controlled simulated environment.

## Discussion

While preliminary technical feasibility and high staff acceptability suggest the system addresses critical safety protocol gaps, these findings require cautious interpretation. Key limitations include small sample size, which precludes definitive claims regarding large-scale clinical efficacy, reliance on simulated settings lacking real-world clinical pressures, and the absence of operational metrics. Moreover, due to manual data collection limitations, granular biometric metrics—such as initial scan attempts, False Rejection Rate, and False Acceptance Rate—were not captured but remain a primary focus for future real-world studies. Furthermore, while staff adoption was high, patient acceptance remains a critical barrier often fueled by data privacy anxieties and opaque storage practices [8]. Future deployments must prioritize transparent communication to foster trust, as structured communication and risk assessments significantly improve staff acceptance and project success [9,10]. Finally, future real-world trials must validate safety improvements by assessing the system against damaged fingerprints, environmental variability, and infrastructure dependencies.

In conclusion, this pilot demonstrates preliminary technical feasibility and user acceptability. While these findings do not measure hard clinical outcomes like workflow efficiency or real-world error reduction, they establish foundational technical reliability, warranting further in vivo studies. Anticipated improvements in patient safety and clinical operations require empirical validation through planned real-world clinical studies.

## Abbreviations

SUS: System Usability Scale
